# Effectiveness and Safety of Talc Slurry Pleurodesis in the Treatment of Patients with Malignant Pleural Effusion and Low Karnofsky Performance Status Scores

**DOI:** 10.3390/jcm14217527

**Published:** 2025-10-23

**Authors:** Eliza Kastrati, Antigona Hasani, Fadil Gradica, Tefta Isufaj Haliti, Aidana Bolat, Ilir Hoxha

**Affiliations:** 1 Chest Department, University Clinical Centre of Kosovo, 10000 Prishtina, Kosovo; kastratieliza@hotmail.com; 2 Department of Anaesthesiology, Faculty of Medicine, University of Hasan Prishtina, 10000 Prishtina, Kosovo; antigona.hasani@uni-pr.edu; 3 University City Hospital of Tirana “Shefqet Ndroqi”, 1044 Tirana, Albania; fadil_gradica68@hotmail.com; 4 Clinic of Obstetrics and Gynaecology, University Clinical Centre of Kosovo, 10000 Prishtina, Kosovo; 5 Evidence Synthesis Group, 10000 Prishtina, Kosovo; aidana.bolat.gr@dartmouth.edu (A.B.); ilir@evidencesynthesis.group (I.H.); 6 The Dartmouth Institute for Health Policy and Clinical Practice, Geisel School of Medicine at Dartmouth, Lebanon, NH 03766, USA; 7 Department of Health Management, Heimerer College, 10000 Prishtina, Kosovo

**Keywords:** malignant pleural effusion (MPE), talc slurry pleurodesis, Karnofsky Performance Status (KPS) score, neutrophil-to-lymphocyte ratio (NLR), pleurodesis

## Abstract

**Background/Objectives**: Malignant pleural effusion is a common and distressing complication of advanced cancer, often resulting in severe symptoms such as dyspnoea, which significantly impacts patients’ quality of life. This study evaluates the effectiveness and safety of talc slurry pleurodesis as a palliative treatment for recurrent malignant pleural effusion and examines predictive factors for pleurodesis success and survival. **Methods**: A single-centre cohort study was conducted at the Thoracic Surgery Department of the University Clinical Centre of Kosovo between April 2022 and March 2024. The study included adult patients with recurrent and symptomatic malignant pleural effusion who met specific inclusion criteria for performing pleurodesis. Patients were followed prospectively with routine clinical evaluations until the conclusion of the study on 9 September 2024. Descriptive and inferential statistics were employed to evaluate the success of pleurodesis and its associated outcomes. **Results**: The study shows a success rate of 84.6% at 30 days post-procedure, with 57.6% achieving complete success and 42.4% partial success. Talc slurry pleurodesis was associated with minimal complications, with chest pain and fever being the most common adverse effects. The most significant predictors of survival post-pleurodesis identified were the Karnofsky Performance Status score and serum neutrophil-to-lymphocyte ratio. Patients with higher Karnofsky Performance Status scores and lower neutrophil-to-lymphocyte ratios had improved survival outcomes. **Conclusions**: This study suggests that talc slurry pleurodesis is an effective and safe option for managing malignant pleural effusions in patients with low-performance status, offering symptom relief and potentially extending survival in certain patients. Further research is required to refine predictive models for treatment success.

## 1. Introduction

Malignant pleural effusion (MPE) occurs in individuals with disseminated malignant disease [1,2] and can arise from malignancies of various origins. Lung and breast cancer are the leading causes [3,4,5], accounting for approximately 50-65% of all cases [6,7]. MPE is one of the most common and debilitating conditions in advanced malignancies. Dyspnoea, being the most prevalent clinical symptom, is succeeded by chest pain, dry cough, and reduced physical activity [7,8,9,10]. MPE is associated with a poor prognosis and a significant subsequent decline in quality of life. The median survival of patients following the diagnosis of malignant pleural effusion (MPE) typically ranges from 3 to 12 months [6,11].

The primary goal of treatment is to manage symptoms by controlling pleural effusion, thereby enabling a better quality of life. Various treatment strategies have been developed to address MPE. They include therapeutic thoracentesis, pleurodesis, and the use of indwelling pleural catheters (IPC). However, the choice of the most suitable therapeutic intervention is influenced by several factors, including expected survival, tumour type, patient performance status, and the extent of lung re-expansion following fluid drainage [6,12].

The British Thoracic Society guidelines recommend that therapeutic thoracentesis remain a suitable treatment option for patients with a life expectancy of less than one month [12]. However, recurrent MPE should be managed with long-term palliative treatments, such as pleurodesis or IPC, for patients with a life expectancy longer than one month [12]. It is worth noting that pleurodesis is often regarded as the most effective palliative treatment for recurrent MPE in clinical practice [7,10,11,13].

Pleurodesis is a procedure that induces pleural inflammation to create adhesions between the visceral and parietal pleura. This procedure is commonly performed through chemical sclerosants or mechanical abrasion [8]. By closing off the pleural space, pleurodesis prevents further accumulation of pleural fluid. Several chemical agents, such as bleomycin, tetracycline, talc, and doxycycline, could be utilised for the procedure. However, talc remains internationally regarded as a sclerosant due to its confirmed safety, efficacy, and relatively low cost [12]. Although the treatment efficacy typically ranges from 70% to 100% [9,14], the procedure has possible risks, including fever and chest pain as side effects, as well as acute respiratory distress syndrome (ARDS), which is a serious complication. The possibility of this complication may be influenced by factors such as the dosage and particle size of talc [15].

Talc slurry pleurodesis is commonly used for chemical pleurodesis in long-term palliative treatment for malignant pleural effusion (MPE) at the Thoracic Surgery Department of the University Clinical Centre of Kosovo. Two primary methods for intrapleural talc administration incorporate chest tube “Talc slurry” pleurodesis and thoracoscopic “Talc poudrage”, which are equally successful and safe in the treatment of MPE [7,15,16]. It should be highlighted that talc slurry pleurodesis is preferred over thoracoscopic talc poudrage due to our extensive experience with the technique. Also, in cases where pleurodesis fails, since the indwelling pleural catheters (IPC) are not available, treatment will continue with repeated thoracocentesis for symptom control or, rarely, with a second attempt of pleurodesis if the lung is expandable and the patient is fit for another attempt. The data from Kosovo were used to examine the safety and efficacy of talk slurry pleurodesis, with additional aims of identifying predictors of treatment outcomes and evaluating the survival benefits associated with successful pleurodesis in patients. This study is the first notable study of the treatment of MPE in patients from Kosovo.

## 2. Methods

### 2.1. Study Design

The prospective cohort study was conducted at the Thoracic Surgery Department of the University Clinical Centre of Kosovo between April 2022 and March 2024. The duration of two years corresponded to the expected caseload of patients meeting the inclusion criteria for pleurodesis, allowing for comprehensive enrolment and follow-up. This study was conducted and reported in accordance with the STROBE (Strengthening the Reporting of Observational Studies in Epidemiology) guidelines to ensure transparent and comprehensive reporting of observational research [17].

### 2.2. Participants

The study included adult patients with recurrent and symptomatic MPE who had a confirmed diagnosis of MPE through pleural fluid cytology or a CT-guided pleural biopsy, experienced symptomatic improvement following thoracocentesis, had a Karnofsky Performance Status (KPS) score of ≥40, and had a predicted survival of greater than one month. Patients were excluded from the study in case of incomplete lung expansion after chest tube drainage, as indicated by a post-drainage chest radiograph showing lung re-expansion of less than 90%, pleural fluid pH < 7.20, previous pleurodesis or chest tube drainage and administration of systemic chemotherapy within two weeks before or during the first 30-day period following pleurodesis.

### 2.3. Talc Slurry Pleurodesis Procedure

Chest tube insertion was performed in a minor surgical room. The patient was placed in the supine position, and the surgical area was cleaned and draped in a sterile manner. A local anaesthetic of 10 mL of 2% lidocaine was injected, and a 2 cm incision was made in the fifth or sixth intercostal space along the midaxillary line. A 24-French chest tube was then inserted and appropriately positioned in the pleural cavity for drainage. The chest tube was secured with sutures and connected to an underwater seal. Pleural effusion was initially drained by gravity, followed by suction from a wall-mounted pump set at a pressure of −20 cm H_2_O, if necessary. A posteroanterior chest radiograph was obtained once the daily tube output was less than 150 mL. Pleurodesis was performed if the radiograph showed lung re-expansion of greater than 90% of the affected hemithorax. It is worth noting that traditionally, including in our centre, large-bore chest tubes are used for talc ‘slurry’ pleurodesis under the assumption that they are less likely to become blocked by clots or fibrin. However, studies have not confirmed this assumption [18,19,20]. The British Thoracic Society (BTS) recommends using small-bore chest tubes for pleurodesis to improve patients’ comfort.

The pleurodesis procedure was carried out at the patient’s bedside. Prior to the procedure, intravenous analgesics, including 100 mg of Tramadol hydrochloride and 10 mg of Metoclopramide, were administered with 100 mL of sterile 0.9% saline solution. In accordance with international guidelines recommendations for pleurodesis [12,21,22], 4 g of graded sterile talc (Steritalc™ F4, Novatech, La Ciotat Cedex, France) were used, prepared as a mixture with 100 mL of sterile saline (0.9%) and 10 mL of 2% lidocaine. A talc mixture was installed into the pleural cavity through the chest tube using 50 mL catheter-tip syringes. The chest tube was then clamped for one hour while the patient underwent rotational manoeuvres (rotating 90 degrees every 15 min). After one hour, the chest tube was reopened and connected to suction at −20 cm H_2_O for at least 24 h. When the daily tube output decreased to less than 150 mL/24h, a posteroanterior chest radiograph was taken to confirm complete lung expansion and adequate fluid evacuation. Upon confirmation, the chest tube was removed, and the patient was discharged.

### 2.4. Outcomes

The pleurodesis outcome was assessed radiologically and clinically on the 30th day following the procedure. Outcomes were classified as follows. Complete success, i.e., absence of pleural fluid on chest radiograph and symptom relief until death. Partial success, i.e., reaccumulation of fluid less than 50% of the baseline volume, with the patient remaining asymptomatic and not requiring further thoracocentesis for the remainder of their life. Failed pleurodesis, i.e., radiographic re-accumulation of pleural fluid equal to or more than 50% of baseline volume and/or recurrence of symptoms, effusion-related requiring repeated thoracocentesis; or patients with prolonged large daily output (>150 mL/24 h) for more than 5 days after pleurodesis. Complete and partial success were considered successful outcomes of pleurodesis. Furthermore, we were interested in identifying diverse clinical and biochemical predictors of pleurodesis success and survival.

### 2.5. Data Collection

For each patient, we recorded demographic information (age, sex), KPS score, histopathological type of primary malignancy, affected hemithorax, time from diagnosis of MPE to chest tube drainage, number of previous thoracocenteses, the volume of pleural effusion drained before and after pleurodesis, duration of chest tube before pleurodesis, biochemical measurements of pleural effusion (including pH, LDH, protein, glucose), pleural and serum neutrophil-to-lymphocyte ratios, adverse effects of talc administration, and the date of any adverse events or death.

All patients underwent a comprehensive pre-treatment evaluation, which included a detailed medical history (including history of malignant disease, malignant pleural effusion and other comorbidities), physical examination, laboratory tests (including complete blood count, coagulation profile, and renal and hepatic function tests), pleural fluid analysis for biochemical parameters, a baseline posteroanterior chest radiograph before drainage, and an assessment of KPS based on the results of the clinical evaluation. Pleural tapping was usually performed one day before the procedure, and 20 mL of pleural fluid was collected for biochemical analysis (LDH, proteins, glucose, neutrophil, and lymphocyte counts). The pH of pleural fluid was measured within 60 min after tapping, using a blood pH/gas analyser (GEM Premier 3500, Instrumentation Laboratory Company, 180 Hartwell Road, Bedford, MA, USA), with a 2 mL sample of pleural fluid collected in a heparinised syringe.

Patients were followed prospectively with routine clinical evaluations and chest radiographs during ambulatory visits. The first follow-up visit was scheduled for the fifth day after discharge, with a second visit scheduled for the 30th day after pleurodesis. Subsequent visits occurred monthly for the first three months and then every three months thereafter until the patient’s death or the study’s conclusion on 9 September 2024. Patients were advised to return for unscheduled visits if they experienced a recurrence of symptoms or other complications. Patients were contacted by telephone two days before each scheduled visit; if there was no response, their close relatives were contacted. Patients who missed visits were considered lost to follow-up and excluded from the study.

### 2.6. Statistical Analysis

Descriptive statistics were initially employed to examine the distribution of cancer types in the study sample, including the number of cases and their respective proportions. A descriptive analysis of relevant variables, including patient demographics, clinical status, and biochemical parameters, was conducted for the entire sample and subgroups stratified by pleurodesis success. Binary variables are reported as counts and percentages, normally distributed variables as means with standard deviations, and non-normally distributed variables as medians with interquartile ranges. The chi-square test for binary variables, the *t*-test for normally distributed continuous variables and the Mann–Whitney U test for non-normally distributed continuous variables were used to understand the differences between subgroups.

To evaluate how various biomarkers and clinical factors predict the success of pleurodesis, a receiver operating characteristic (ROC) analysis was performed. Survival rates between subgroups based on pleurodesis success were compared through survival analysis. The median survival times were calculated, and the log-rank test was used to assess differences between the groups. Survival time was measured from the date of the pleurodesis procedure until the patient’s death or the end of the study period, whichever occurred first. To identify factors influencing survival outcomes, Cox regression with robust variance was applied in crude and adjusted models. The biomarkers assessed included serum and pleural fluid biochemical parameters, such as lactate dehydrogenase (LDH), total protein, glucose, pH, and pleural fluid cellular counts (neutrophils and lymphocytes), which served as objective indicators of local inflammatory and metabolic activity. The predictors of survival were identified in existing published literature. Variables were included in adjusted models if they showed an effect with a *p*-value lower than 0.25 in the crude model. Continuous predictors were converted to binary variables using the median value as the benchmark. All statistical analyses were performed using Stata/BE 18.0 (StataCorp LLC, College Station, TX, USA).

### 2.7. Ethical Considerations

This study involves human participants and was approved by the Ethics Committee of the Faculty of Medicine at the University of Pristina, as well as the Ethics Committee of the Kosovo Medical Chamber, with number 56/2022, dated 12 April 2022. Participants gave informed consent to participate in the study.

### 2.8. Patient and Public Involvement

Patients and the public were not involved in the design, conduct, or reporting of this study. The development of the research question and study design was informed by the patient’s previous clinical experience, as well as the researchers’ involvement in the execution of this study.

## 3. Results

### 3.1. Study Population

**Figure 1:**
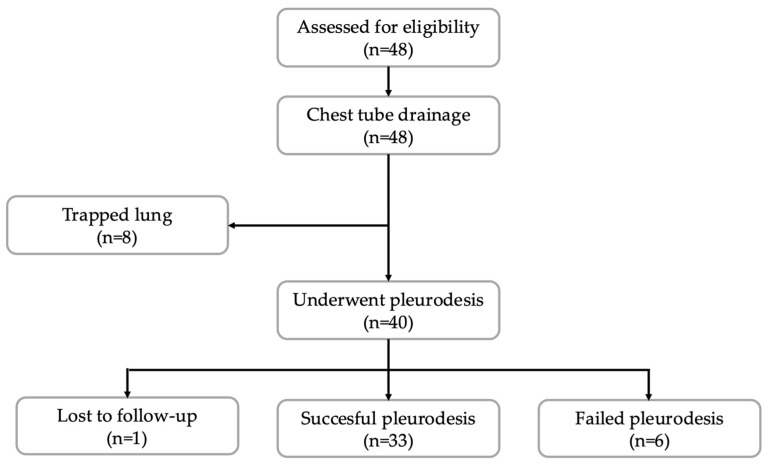
Flowchart of study participants: inclusion and exclusion process.

Between April 2022 and March 2024, a total of 48 patients met the study’s inclusion criteria (Figure 1). All patients underwent chest tube insertion for the drainage of pleural effusions. Of these, eight patients were excluded because they experienced incomplete lung re-expansion, i.e., trapped lung after drainage of the pleural effusion, which prevented them from undergoing pleurodesis. One patient was lost to follow-up. This resulted in a population of 39 patients with MPE and a mean follow-up time of 8.20 ± 8.47 months. Lung cancer was the most common diagnosis, found in 14 patients (35.9%). This was followed by breast cancer in 13 patients (33.3%) (Table 1). Gynaecological cancer was the third most common, diagnosed in three patients (7.7%). Bone cancer, gastrointestinal cancer, haematological cancer and mesothelioma occurred in two patients (5.1%) each. Renal cell carcinoma was diagnosed in a single patient (2.6%).

### 3.2. Baseline Characteristics

The mean age of the patients was 61 years (range 56–68), and the study cohort included 13 males (33.3%) and 26 females (66.6%) (Table 2). Twenty-two patients (56.4%) had right-sided MPE, while 17 (43.6%) had left-sided MPE. The median time from MPE diagnosis to chest tube drainage was 30 days, and the median volume of pleural effusion drained before pleurodesis was 2600 mL. For the talc pleurodesis procedure, the median duration of drainage prior to pleurodesis was eight days, with a median volume of pleural effusion drained post-pleurodesis of 200 mL. Twenty patients (51.3%) had previously undergone more than two thoracocenteses (3–9 procedures) before pleurodesis, while 19 patients (48.7%) had undergone one or two initial thoracocenteses. Pleural fluid testing showed a mean pH of 7.43, a median lactate dehydrogenase (LDH) level of 409 U/L, a median glucose level of 5.4 mmol/L, and a median protein level of 38.1 g/L. The median neutrophil-to-lymphocyte ratio (NLR) in pleural effusion was 0.163, while in serum, it was 3.6. The median Karnofsky Performance Status score of the patients was 50.

### 3.3. Pleurodesis Outcomes

At 30 days following pleurodesis, 33 of the 39 patients (84.6%) had a successful response to talc slurry pleurodesis, while six patients (15.4%) did not achieve a successful outcome (Table 2). Among the 33 patients in the successful group, 19 (57.6%) achieved complete success, and 14 (42.4%) had partial success. The six patients who did not respond to pleurodesis were classified as failed pleurodesis. Two of these six patients experienced prolonged large daily output after the procedure. These patients were discharged with a chest tube connected to a Heimlich valve and a bag for long-term drainage, continuing until the daily output decreased to less than 150 mL/24 h. Four patients in the failure group exhibited significant pleural effusions on chest radiographs, which exceeded 50% of their baseline volume with the need for thoracocentesis, during their 30-day follow-up visit post-pleurodesis. Unfortunately, all four of them needed further treatment of pleural effusion, and they were treated with repeated thoracocentesis due to their poor performance status. As a result, no deaths occurred within the 30 days following the pleurodesis procedure. Chest pain and fever were the only side effects observed, both occurring within 24 h of talc administration. Pain was present in 41% of all cases post-pleurodesis, while fever was present in 10.3%.

Table 2 also summarises the characteristics of the study population, stratified by pleurodesis success and failure. Patients in the pleurodesis success group were significantly older, with a median age of 64 years (IQR 57–69), compared to those in the failure group, whose median age was 57 years (IQR 47–60, *p* = 0.035). There were no significant differences between the groups in terms of gender distribution, side of hemithorax affected (right vs. left), KPS score, or the number of previous thoracocenteses. Although not statistically significant, patients in the pleurodesis failure group had a larger volume of pleural effusion drained before pleurodesis (3150 mL, IQR 1900–5100) compared to those in the success group (2550 mL, IQR 1850–4400, *p* = 0.508). Similarly, the effusion volume drained post-pleurodesis was larger in the failure group (725 mL, IQR 100–2000) compared to the success group (200 mL, IQR 100–550, *p* = 0.308). Time from diagnosis of MPE to chest tube drainage, LDH, glucose, pH, and protein levels in pleural effusion demonstrated no significant associations with pleurodesis outcome. Remarkably, the neutrophil-to-lymphocyte ratio (NLR) in pleural effusion was elevated in the failure group (median 0.26, IQR 0.19–0.53) when compared with the success group (median 0.16, IQR 0.05–0.46), although the difference did not reach statistical significance (*p* = 0.150). Similarly, the NLR in serum was slightly higher in the failure group (median 4.11, IQR 3.48–12.10) relative to the success group (median 3.53, IQR 2.78–5.94, *p* = 0.311).

### 3.4. Predictors of Success of Pleurodesis

Testing for potential predictors of pleurodesis success, including pleural fluid pH, LDH, glucose, protein levels, and the neutrophil-to-lymphocyte ratio (NLR) in both serum and pleural fluid, demonstrated no significant sensitivity in predicting pleurodesis outcomes except for the Karnofsky Performance Status score. The ROC analysis for the KPS score, using a cutoff point of 60, yielded an Area Under the Curve (AUC) of 0.631 (95% CI: 0.359–0.904), indicating a modest discriminative ability. At this threshold, the sensitivity was 54.55%, and the specificity was 83.33%, demonstrating the test’s moderate ability to correctly identify positive cases while maintaining a high level of specificity. The positive predictive value (PPV) was 94.74%, and the negative predictive value (NPV) was 25.00%, resulting in an overall correct classification rate of 58.97%.

### 3.5. Survival Analysis

**Figure 2:**
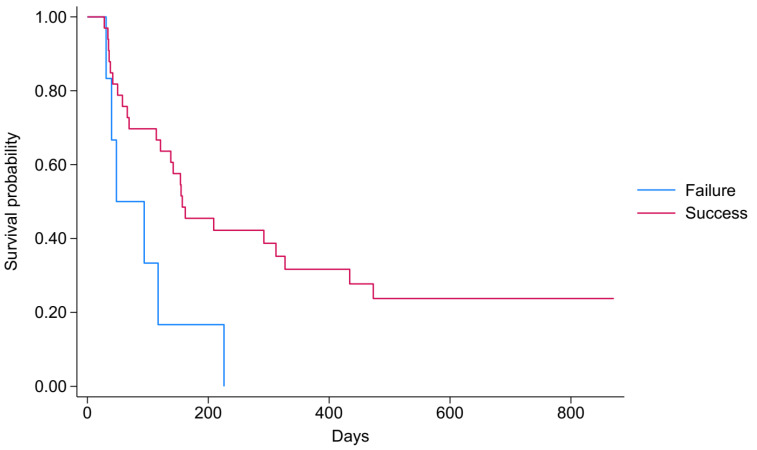
Survival analysis by the success of pleurodesis.

Nine patients were alive at the end of the study, i.e., by the time follow-up was completed on 9 September 2024. Fourteen patients (35.9%) had died by the end of the first three months, and 23 patients (58.97%) had died by the end of the sixth month. The Kaplan–Meier survival curve for pleurodesis outcomes was used to detect distinct survival trajectories between the “Failure” and “Success” groups (Figure 2), which indicated more favourable results for patients in the “Success” group. The findings are explained by the “Success” group consistently maintaining a higher survival probability in contrast to the “Failure” group. The “Failure” group’s survival probabilities decrease more rapidly, signifying a higher rate of adverse events or mortality over the observed period. The median survival time was 157 days for the “Success” group, while it was only 48 days for the “Failure” group. The log-rank test, used to compare survival between the “Failure” and “Success” groups in relation to pleurodesis outcomes, indicated a statistically significant difference in survival functions (*p* = 0.017).

### 3.6. Predictors of Survival After Pleurodesis

Table 3 outlines the predictive factors for survival following talc slurry pleurodesis for three months and at the end of the study period, i.e., 9 September 2024. In the analysis of three-month mortality following pleurodesis, higher Karnofsky Performance Status scores (above the median) were significantly associated with improved survival. Specifically, the unadjusted hazard ratio (HR) was 0.14 (95% CI: 0.03–0.64; *p* = 0.011), and this association remained after adjustment for covariates (adjusted HR = 0.12; 95% CI: 0.03–0.57; *p* = 0.008). Elevated lactate dehydrogenase (LDH) levels showed a trend toward poorer survival (unadjusted HR = 0.34; 95% CI: 0.09–1.24; *p* = 0.103), which became attenuated and non-significant in the adjusted model (adjusted HR = 0.47; 95% CI: 0.13–1.74; *p* = 0.256). No statistically significant associations were observed for pH or glucose levels, as both variables yielded non-significant *p*-values in unadjusted analyses and did not enter the adjusted models. In the unadjusted analysis of end-of-study survival, a Karnofsky Performance Status score greater than the median was associated with a significantly reduced risk of mortality (HR = 0.35, 95% CI = 0.17–0.74, *p* = 0.006). Additionally, an elevated neutrophil-to-lymphocyte ratio (NLR) in serum (above the median) was associated with a greater risk of mortality (HR = 4.00, 95% CI = 1.85–8.68, *p* < 0.001). In the adjusted model, the Karnofsky Performance Status Score persisted as an independent predictor of improved survival (HR = 0.24, 95% CI: 0.10–0.55, *p* = 0.001), while a higher serum NLR continued to be highly correlated with a greater risk of mortality (HR = 5.48, 95% CI: 2.25–13.33, *p* < 0.001).

## 4. Discussion

### 4.1. Summary of Results

The results of this study demonstrate a 30-day post-procedure success rate of 84.6%, with 57.6% of patients achieving complete success and 42.4% partial success. Talc slurry pleurodesis was well tolerated, with minimal complications, including chest pain and fever as the most common side effects. Key predictors of survival after pleurodesis were the Karnofsky Performance Status score and serum neutrophil-to-lymphocyte ratio, with higher KPS scores and lower NLR associated with improved survival.

### 4.2. Strengths and Limitations

This study was conducted in a single-centre setting, which limits the generalisability of the findings to other settings or broader populations. Furthermore, the small cohort size restricts the statistical power of our analysis. In particular, the non-significant associations observed for NLR and several biochemical markers should be interpreted with caution, as they may reflect limited power rather than a genuine lack of association. The wide confidence intervals around these estimates further underscore the reduced precision. While our findings provide valuable exploratory insights, validation in larger cohorts is warranted to confirm these observations. It should also be noted that patients with trapped lungs were not thoroughly evaluated or followed up after being excluded from the study, which could have provided valuable insights into their management and prognosis. The ROC analysis was performed to determine the predictive value of the KPS score. A cutoff of 60 yielded a moderate discriminative ability, suggesting that while the KPS score can provide helpful information, it should be used with caution when predicting pleurodesis outcomes, given the small cohort size and marginal AUC. The strengths of this study include its prospective design, which minimises recall bias and permits systematic data collection. The inclusion and exclusion criteria, significant for internal validity, ensure a homogeneous study population. The use of standardised procedures for pleurodesis, chest tube insertion, and follow-up assessments minimises inconsistencies in treatment delivery. Finally, the comprehensive data collection, which includes demographic, biochemical, clinical, and radiological parameters, enables an in-depth analysis of predictors of pleurodesis success and survival. It is also worth mentioning that although chest ultrasonography was not used in our procedure protocol, several studies have explored its role in assessing pleurodesis efficacy, particularly by detecting the absence of lung sliding or pleural adherence as early predictors of successful pleurodesis [23,24].

### 4.3. Comparison with Other Studies

A success rate of 84.6% at 30 days post-procedure is in line with success rates observed in a systematic review of talc pleurodesis literature [9,14] as well as other published studies [25,26]. Remarkably, all 33 patients in this study who achieved successful pleurodesis remained asymptomatic and required no further thoracocentesis until their death or the conclusion of the study. This is consistent with the findings of other authors, who noted that successful pleurodesis (either complete or partial) often lasts until the patient’s death [27,28]. Our results of talc pleurodesis demonstrate that talc is a more effective agent than doxycycline (62.5%), tetracycline (76%) and bleomycin (79.1%) when used for pleurodesis in the treatment of MPE [4,29,30].

Several other studies have investigated various clinical and biochemical parameters as predictors of pleurodesis success with conflicting results. In a study by Burgers et al., pleural effusion parameters such as LDH, total protein, and pH did not correlate with pleurodesis success [1]. Instead, systemic antitumor treatment and lung re-expansion after drainage were identified as predictors of success [1]. Arafa et al. found that pH, glucose, Adenosine Deaminase (ADA), and CRP were the only pleural effusion parameters significantly influencing pleurodesis success [31]. Similarly, in a study by Rafei et al., only the pH of pleural effusion and chest tube size were associated with pleurodesis outcomes [2]. Other factors, including LDH and glucose of pleural effusion, did not show statistically significant associations [2]. In contrast, a study by Yildirim et al. found that KPS score and pleural effusion pH, glucose, cholesterol and ADA were significantly correlated with pleurodesis outcomes, with pH and ADA being independent predictors of success [25]. Ak et al. observed significant differences in KPS score, pleural fluid pH, lung re-expansion before the procedure, amount of fluid drained, and chest tube drainage duration, which could impact the success of pleurodesis [10].

When it comes to mortality, Stefani et al. found a similar ratio of deaths in the first three months following talc pleurodesis in a study population of 109 participants [32]. For median survival, our study results are consistent with several previous studies where authors have observed longer survival with successful pleurodesis compared to failed pleurodesis in patients with MPE [10,11,13]. The lower median survival in our study is likely a reflection of a population with low KPS scores. In accordance with our result, another author, in a study population of 64 patients with low KPS scores, observed poor survival [33].

When it comes to predictive factors of survival, our results are consistent with several studies. In a systematic review by Hassan et al., performance status was identified as one of the most significant factors influencing survival in patients with MPE undergoing pleurodesis [13]. Burrows et al. found that the KPS score is the only predictive factor of survival [34]. Performance status, WBC, and NLR in serum were predictors of survival in a study reported by Anevlaviset et al. [35].

### 4.4. Clinical and Research Implications

Talc slurry pleurodesis is one of two methods for dealing with MPL. Talc slurry pleurodesis provides a definitive option by obliterating the pleural space, while indwelling pleural catheters (IPC) allow outpatient management with symptom relief, flexibility, and reduced hospital stays. Talc slurry pleurodesis has demonstrated high effectiveness, achieving an 84.6% success rate with minimal complications, which supports its use as a treatment for MPE in patients with a low KPS score. Up to 32% of carefully selected patients for pleurodesis do not survive 30 days, and this limits the value of available survival predictive factors [9,15,36]. Among available factors, the performance status score represents the most predictive indicator [9,34]. Our study identified the KPS score and serum neutrophil-to-lymphocyte ratio as significant predictors of survival. Pleurodesis with favourable results was associated with prolonged survival, highlighting its role in managing symptoms beyond the initial treatment. It is worthwhile to continue assessing the predictive factors of pleurodesis outcomes and identifying predictors of survival to more effectively select patients for pleurodesis and increase the procedure’s success rate. Until then, pleurodesis should be offered more frequently and early on to patients with MPE. Further research is necessary to improve predictive models, refine treatment selection criteria, and advance personalised treatment strategies for MPE by considering individual patient characteristics and clinical determinants.

## 5. Conclusions

Talc slurry pleurodesis is an effective, safe local treatment with long-lasting success for patients with low KPS scores and should be considered for every eligible patient with MPE, except for those who are terminally ill, provided there is satisfactory lung re-expansion. None of the investigated predictors could accurately predict the success of pleurodesis. We observed longer survival in patients who achieved successful talc pleurodesis compared to those who failed to achieve pleural symphysis, suggesting a possible survival benefit for patients in the successful group. Additionally, we identified the KPS score and NLR in serum as significant predictors of survival in patients with MPE undergoing talc slurry pleurodesis. Nevertheless, when selecting patients for pleurodesis, it is essential to remember that the primary goal of talc slurry pleurodesis is to improve quality of life by managing pleural effusion rather than prolonging survival. Future research is needed to explore further and identify reliable predictors of pleurodesis success, ultimately improving patient selection and clinical outcomes.

## Figures and Tables

**Table 1:** Diagnosis of primary cancer.

Cancer Diagnosis		All (n = 39)
		n (%)
	Bone cancer	2 (5.13)
	Breast cancer	13 (33.33)
	Gastrointestinal cancer	2 (5.13)
	Gynaecological cancer	3 (7.69)
	Haematological cancer	2 (5.13)
	Lung cancer	14 (35.90)
	Mesothelioma	2 (5.13)
	Renal cell cancer	1 (2.56)

**Table 2:** Characteristics of the study population.

Characteristics	All (n = 39)	Pleurodesis Failure (n = 6)	Pleurodesis Success (n = 33)	
	n (%)	n (%)	n (%)	*p* Value
Age, years **	61 (56–68)	57 (47–60)	64 (57–69)	0.035
Gender, male vs. female	13 (33.3)	3 (50.0)	10 (30.3)	0.346
The left side is affected	17 (43.6)	2 (33.3)	15 (45.5)	0.582
KPS score **	50 (50–60)	50 (50–50)	60 (50–60)	0.290
Two or less thoracocentesis	19 (48.7)	3 (50.0)	16 (48.5)	0.946
Number of drainage days pre-pleurodesis, days **	8 (6–11)	8.5 (8–10)	7 (6–11)	0.624
Volume of effusion drained before pre-pleurodesis, mL **	2600 (1900–4500)	3150 (1900–5100)	2550 (1850–4400)	0.508
Volume of effusion drained post-pleurodesis, mL **	200 (100–600)	725 (100–2000)	200 (100–550)	0.308
Time from diagnosis of MPE to chest tube drainage, days **	30 (14–60)	20 (9–43)	30 (15–60)	0.284
pH *	7.43 (0.114)	7.41 (0.076)	7.43 (0.120)	0.497
LDH, U/L **	409 (240–774)	416 (188–1107)	409 (243–720)	0.876
Glucose, mmol/L **	5.4 (3.4–7.0)	4.75 (3.3–5.4)	5.7 (3.6–7.0)	0.436
Proteins, g/L **	38.1 (33.1–43.0)	34.3 (30.7–41.4)	38.2 (34.6–43.0)	0.471
NLR of serum **	3.6 (2.8–6.1)	4.1 (3.5–12.1)	3.5 (2.8–5.9)	0.311
NLR of pleural liquid **	0.16 (0.06–0.46)	0.26 (0.19–0.53)	0.16 (0.05–0.46)	0.150

* Mean and Standard deviation, ** Median and Inter Quartile Range.

**Table 3:** Prediction factors of survival after pleurodesis.

Three-Month Mortality								
	Unadjusted				Adjusted *			
		95% CI				95% CI		
Characteristics	HR	Lower	Upper	*p* Value	HR	Lower	Upper	*p* Value
KPS score, >median	0.14	0.03	0.64	0.011	0.12	0.03	0.57	0.008
pH, >median	1.07	0.36	3.18	0.906				
LDH, >median	0.34	0.09	1.24	0.103	0.47	0.13	1.74	0.256
Glucose, >median	1.08	0.36	3.20	0.895				
Proteins, >median	0.86	0.29	2.55	0.781				
NLR of serum, >median	5.04	1.38	18.44	0.014	4.82	1.25	18.62	0.023
NLR of liquid, >median	1.99	0.65	6.08	0.229				
**End of Study Period Mortality**								
	**Unadjusted**				Adjusted *			
		**95% CI**				**95% CI**		
**Characteristics**	**HR**	**Lower**	**Upper**	***p*** **Value**	**HR**	**Lower**	**Upper**	***p*** **Value**
KPS score, >median	0.35	0.17	0.74	0.006	0.24	0.10	0.55	0.001
pH, >median	1.16	0.56	2.41	0.681				
LDH, >median	0.55	0.26	1.15	0.110	0.84	0.39	1.83	0.657
Glucose, >median	0.79	0.38	1.63	0.522				
Proteins, >median	0.81	0.39	1.68	0.572				
NLR of serum, >median	4.00	1.85	8.68	<0.001	5.48	2.25	13.33	<0.001
NLR of liquid, >median	1.46	0.71	3.00	0.306				

* Adjusted for the variables present in the model.

## Data Availability

Data are available on reasonable request. The datasets used and analysed during the current study are available from the corresponding author upon reasonable request.
